# Aspirin and P2Y_12_ inhibition attenuate platelet-induced ovarian cancer cell invasion

**DOI:** 10.1186/s12885-015-1634-x

**Published:** 2015-09-09

**Authors:** Niamh M. Cooke, Cathy D. Spillane, Orla Sheils, John O’Leary, Dermot Kenny

**Affiliations:** 1 Department of Molecular and Cellular Therapeutics, Royal College of Surgeons in Ireland, 123 St Stephens Green, Dublin 2, Ireland; 2 The Biomedical Diagnostics Institute, Dublin City University, Dublin, Ireland; 3 Department of Histopathology, Trinity College Dublin, Dublin, Ireland; 4 Pathology Department, Coombe Women’s Hospital, Dublin, Ireland

## Abstract

**Background:**

Platelet-cancer cell interactions play a key role in successful haematogenous metastasis. Disseminated malignancy is the leading cause of death among ovarian cancer patients. It is unknown why different ovarian cancers have different metastatic phenotypes. To investigate if platelet-cancer cell interactions play a role, we characterized the response of ovarian cancer cell lines to platelets both functionally and at a molecular level.

**Methods:**

Cell lines 59 M and SK-OV-3 were used as *in vitro* model systems of metastatic ovarian cancer. Platelet cloaking of each cell line was quantified by flow cytometry. Matrigel invasion chamber assays were used to assess the invasive capacity of the cell lines. The induction of an EMT was assessed by morphology analysis and by gene expression analysis of a panel of 11 EMT markers using TaqMan RT-PCR.

**Results:**

SK-OV-3 cells adhered to and activated more platelets than 59 M cells (*p* = 0.0333). Platelets significantly promoted the ability of only SK-OV-3 cells to invade (*p ≤* 0.0001). Morphology and transcritpome analysis indicated that platelets induce an epithelial-to-mesenchymal transition phenotype in both cells lines, with a more exaggerated response in SK-OV-3 cells. Next, we investigated if antiplatelet agents could abrogate the platelet-induced aggressive phenotype in SK-OV-3 cells. Both aspirin (*p ≤* 0.05) and 2-methylthioadenosine 5′-monophosphate triethylammonium salt hydrate (P2Y_12_ inhibitor; *p ≤* 0.01) significantly decreased their invasion capacity, and effectively reverted invasion to levels comparable to SK-OV-3 cells alone.

**Conclusion:**

While there is increasing evidence for the cancer-protective effect of aspirin, this study suggests P2Y_12_ inhibition may also play a role. Understanding these complex interactions between platelets and cancer cells could ultimately allow the establishment of therapies tailored to inhibiting metastasis, thus significantly reducing cancer morbidity.

## Background

A growing body of evidence suggests that platelets have a key role in successful haematogenous metastasis. Following initial contact, the formation of tumour cell-induced platelet aggregates (TCIPA) facilitate immune evasion [1, 2], provide angiogenic and growth factors [3, 4], and also promote microvascular arrest of at distant sites [5–7]. Experimental blockade or deletion of platelet receptors interferes with the TCIPA process, resulting in significantly decreased metastasis [8, 9]. Additionally, similar results are observed upon induction of thrombocytopenia in mice, an effect reversed by transfusion of platelet rich plasma [10, 11].

The ability of circulating tumour cells (CTCs) to sustain the epithelial-mesenchymal transition (EMT) is essential for cancer progression [12]. Many growth factors and cytokines secreted from both stromal cells and blood components contribute to this process, including epidermal growth factor, insulin-like growth factor, and hepatocyte growth factor as well as matrix metalloproteinases and urokinase plasminogen activator [13, 14]. Platelets also provide key instructive signals that affect tumour cell invasiveness and metastatic potential. Using mouse cells lines and models, Labelle *et al.* have demonstrated that platelet-derived transforming growth factor β (TGF-β) along with direct platelet-tumour cell contact can induce EMT in tumour cells *in vitro*, and also enhance their extravasation and seeding *in vivo* [15]. Moreover, a recent study demonstrated a direct proliferative effect of platelets on ovarian cancer cells mediated via TGF-β and partially dependent on platelet signalling through cyclooxygenase-1 (COX-1) [16].

Ovarian cancer has the highest mortality rate of all gynaecological malignancies and is the fifth leading cause of all cancer-related deaths in women [17]. About 200,000 cases of ovarian cancer occur worldwide each year. Over 70 % of ovarian cancer patients present with advanced stage III and IV disease, which is associated with a poor prognosis and high mortality rate [18]. Recent studies have demonstrated that ovarian cancer patients have an abundance of CTCs in their blood [19, 20]. Moreover these studies have identified ovarian cancer cells at distant sites, including the liver, spleen and bone aspirates [21–23]. The biological mechanism for hematogenous dissemination of ovarian cancer remains poorly understood.

We have described a potent dynamic interaction between platelets and ovarian cancer cells *in vitro*, which results in pro-survival and pro-angiogenic signalling in the cancer cells [24]. In this current study, we sought to further our understanding of this relationship by characterising the interactions between platelets and ovarian cancer cells. We investigated the effect of platelet adhesion to two ovarian cancer cell lines, and how this communication can mediate platelet activation and alter ovarian cancer cell EMT expression profile, morphology and invasive behaviour. Since the antiplatelet agent aspirin is associated with a reduce risk of death and distant metastasis in cancer patients [25–27] including those with ovarian cancer [28], we investigated the effect of aspirin and the antiplatelet P2Y_12_ blocker, 2-methylthioadenosine 5′-monophosphate triethylammonium salt hydrate (2MeSAMP), on the platelet cancer cell interaction.

## Methods

### Ethics statement

Blood collection was approved by the Royal College of Surgeons in Ireland ethics committee. Healthy female donors who had not taken medications known to affect platelet function for ≥10 days were recruited. Written informed consent was obtained prior to phlebotomy.

### Cell lines

Two metastatic ovarian cancer cell lines were employed in this study, 59 M and SK-OV-3, both originating from the ascites. 59 M cells [European collection of cell cultures, Salisbury, UK] represent an ovarian epithelial carcinoma of endometrioid type with clear cell components. SK-OV-3 cells [American Tissue Culture Collection, Manassas, VA, USA] represent an ovarian epithelial adenocarcinoma. 59 M cells were maintained in Dulbecco’s Modified Eagle Medium and SK-OV-3 cells in McCoy’s 5A media, each supplemented with 10 % fetal bovine serum (FBS), 2 mM L-glutamine, 100 units/ml penicillin, and 100 ug/ml streptomycin. Cells were cultured in standard conditions in a humidified atmosphere containing 5 % CO_2_ at 37 °C.

### Platelet preparation

Whole blood was collected by venipuncture through a 19-gauge butterfly needle. Platelet-rich plasma (PRP) was prepared from 3.2 % trisodium citrated blood (10 % vol/vol) centrifuged at 170 *g* for 10 min. For the preparation of washed platelets, blood was collected into Acid-Citrate-Dextrose (ACD: 38 mM citric acid, 75 mM sodium citrate, 124 mM D-glucose) as anticoagulant (15 % vol/vol) and centrifuged at 170 g for 10 min. PRP was acidified to pH 6.5 with ACD, 1 μM PGE1 was added and centrifuged at 720 g for 10 min. The platelet pellet was resuspended in JNL buffer [130 mM NaCl, 10 mM sodium citrate, 9 mM NaHCO3, 6 mM D-glucose, and 0.9 mM MgCl2, 0.81 mM KH2PO4, and 10 mM Tris, pH 7.4] and supplemented with 1.8 mM CaCl_2_.

### Platelet adhesion assay

Platelet adhesion to ovarian cancer cells was measured by flow cytometry, based on the detection of CD42b (GPIbα) on the surface of cancer cells following co-incubation. Washed suspensions of ovarian cancer cells (1 × 10^6^/ml) were incubated with PRP (1:1000 cancer cell-platelet ratio) for 1 min under low shear on a rocking table (12 oscillations per minute, opm). At this ratio, no tumour cell-induced platelet aggregation is observed, but there is efficient coating of tumour cells by platelets with a degranulated phenotype [29]. Next, samples were washed, fixed with 3.7 % paraformaldehyde, blocked with 1 % BSA and labelled with either allophycocyanin (APC) mouse anti-human CD42b antibody or isotype control (Becton Dickinson). Samples were analysed within 1 h by flow cytometry (Becton Dickinson). Using a log forward scatter versus log side scatter dot plot, a two dimensional analysis gate was drawn around the cancer cell population, and a fluorescence histogram was obtained for 10,000 events for each sample. Platelet aggregates and cancer cells duplets were excluded using size based gating. Data was analysed using BD FACS DIVA^™^ software. The percentage of platelet tumour cell adhesion was calculated as the percentage of cells within the tumour cell gate positive for the platelet specific marker CD42b relative to the isotype control.

### Platelet activation assay

Platelet activation by ovarian cancer cells was measured by flow cytometry, based on the detection of P-selectin (CD62P) on the surface of platelets following co-incubation. P-selectin is stored internally in alpha-granules of resting platelets and is translocated to the surface upon activation. Washed suspensions of cancer cells (1 × 10^6^/ml) were incubated with PRP (1:30 cancer cell-platelet ratio) for 15 min under low shear conditions on a rocking table (12 opm). The reaction was terminated with 1 ml of JNL buffer. Samples were processed as described above and labelled with either APC mouse anti-human P-selectin antibody or isotype control (Becton Dickinson). Samples were analysed as above, gating on the platelet population, and a fluorescence histogram was obtained for 10,000 events for each sample. The percentage of tumour cell induced platelet activation was calculated as the percentage of P-selectin positive platelets relative to the isotype control.

### Platelet inhibitor preparation

Where indicated, cancer cell suspensions or washed platelet suspensions were treated with either 2MeSAMP, an adenosine-based P2Y_12_ antagonist, or aspirin, a COX-1 antagonist. 2MeSAMP was dissolved in water, while aspirin was dissolved in 10 % dimethyl sulfoxide (DMSO). Inhibitor incubation times were 15 min for 2MeSAMP (50 μM) and 30 min for aspirin (20 μM) at 37 °C.

### Platelet aggregation

Donors were verified as aspirin- and 2MeSAMP-responders prior to inclusion in the study. Platelet aggregation was assessed at 37 °C with continuous stirring using a PAP-4/8 aggregometer (Bio-Data Corporation). Aggregation was induced with 20 μM adenosine diphosphate (ADP) or 500 μg/ml arachidonic acid (Bio-Data Corporation), and compared to platelets pre-treated with 2MeSAMP (50 μM) and ASA (20 μM). Platelet aggregation was expressed as the maximal percent change in light transmittance from baseline, using platelet poor plasma as reference. Responsiveness was defined as the relative platelet inhibition induced by the addition of either antiplatelet agent. Relative inhibition (RI) = [(pre-treatment aggregation–post-treatment aggregation)/(pre-treatment aggregation)] × 100 %. Responders were defined as those with a RI of ≥70 %.

### Cancer cell invasion assay

BioCoat® Matrigel® invasion chambers (8-μm pore size) and corresponding control plates (BD Biosciences) were used to examine the effect of platelets on cancer cell invasion. Ovarian cancer cells were incubated with media alone or washed platelets or drug treated washed platelets (final cancer cell-platelet ratio 1:1000) for 24 h. After incubation, suspensions were prepared in serum-free medium (5 × 10^4^ cells/ml) and allowed to invade through Matrigel® and control chambers for 16 and 24 h at 37 °C in 5 % CO_2_. Bottom wells contained 10 % FBS as a chemoattractant. Invaded cells were fixed (10 % formalin for 24 h) and fluorescently labelled (fluoroshield with DAPI). Filters were imaged using an epifluorescence inverted microscope (Zeiss Axiovert-200) and quantified with Image J (NIH). The number of cells per 40× high power field (cells/hpf) was determined in 3 random hpf, and the mean number of cells/hpf was calculated for each membrane. All experiments were carried out with a minimum of three wells per condition. Data are expressed as percentage invasion through Matrigel® chambers relative to the control.

### RNA extraction

Ovarian cancer cells were incubated with media alone, washed platelets or washed platelets pre-treated with antiplatelet agent (cancer cell-platelet ratio 1:1000) for 24 h. After incubation, total RNA was extracted from the samples using the miRVana Kit (Life Technologies), according to the manufacturer’s protocol.

### Real time PCR

The mRNA expression level of 11 EMT related genes was profiled using a custom designed RT-PCR-based TaqMan OpenArray® Panel (Life Technologies) following the manufacturer’s instructions. Briefly, cDNA template was prepared from 2.5 μg of total RNA using the high capacity cDNA archive kit (Life Technologies). The samples were next subjected to real time-PCR analysis using TaqMan mRNA primers and probes on the TaqMan OpenArray platform. Ct (crossing threshold) values for each mRNA target was calculated using the OpenArray® Real-Time qPCR Analysis Software. Expression values were calculated using the comparative Ct method, with beta 2 microglogublin (B2M) used as the endogenous control.

## Results

### Platelet interactions with ovarian cancer cells are heterogeneous

**Fig. 1:**
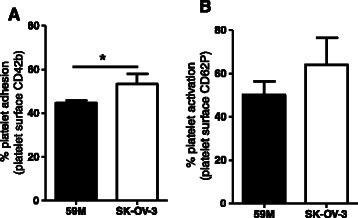
Platelet interactions with ovarian cancer cells are significantly heterogeneous. **a** Platelet adhesion to 59 M (*black bar*) and SK-OV-3 (*white bar*) cells was quantified based on the fluorescence detection of CD42b positive platelets (*n* = 3). *p = 0.0333 was determined by Student’s *t* test. **b** Platelet activation by 59 M (*black bar*) and SK-OV-3 (*white bar*) cells was quantified based on the fluorescence detection of P-selectin (CD62P) positive platelets (*n* = 3). Data shown are mean values + SD

59 M and SK-OV-3 cells were incubated with an excess of platelets under low shear conditions to assess their ability to adhere to and to activate platelets. The degree of platelet adhesion or ‘cloaking’ was measured based on the fluorescent detection of labelled platelets on the surface of cancer cells post incubation, while platelet activation was measured based on the fluorescent detection of platelet surface P-selectin. The percentage of SK-OV-3 cells cloaked with platelets was significantly greater compared to 59 M cells (Fig. 1a. 53 % versus 45 %, *p* = 0.0333). SK-OV-3 cells also induced a higher degree of platelet activation compared to 59 M cells (64 % versus 50 %, Fig. 1b).

### Platelets induce an EMT-like morphology and gene expression profile in ovarian cancer cells

**Fig. 2:**
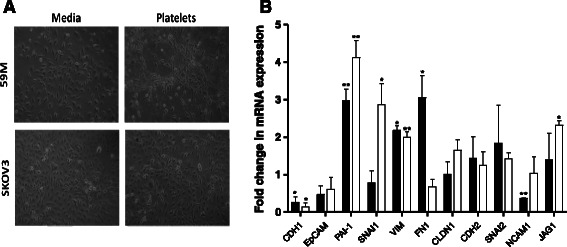
Platelets induce a mesenchymal-like state in ovarian cancer cells. **a** Phase-contrast micrographs of 59 M and SK-OV-3 cells grown in the absence (media) or presence of platelets for 24 h. **b** Results are expressed as fold change in mRNA expression in 59 M (*black bar*) or SK-OV-3 (*white bar*) cells in the presence of platelets relative to control cells grown in the absence of platelets for 24 h (*n* = 3). Values are normalised to B2M (beta 2 microglobulin). Data shown are mean value + SD. **p ≤ 0.01, *p ≤ 0.05 was determined by Student’s *t* test

We have previously demonstrated that the interaction between ovarian cancer cells and platelets affected EMT-related genes [24], a pivotal process in the metastatic cascade. To further these observations, we investigated whether EMT-like changes were induced by platelets in both the SK-OV-3 and 59 M cell lines, and assessed if the levels of induction correspond to the levels of platelet-tumour cell interactions. SK-OV-3 and 59 M cells were co-cultured with platelets for 24 h prior to analysis. Morphological alterations, including loss of cell-cell contact and elongation of shape, indicative of a more mesenchymal like phenotype were observed with both cell lines when co-cultured with platelets for 24 h (Fig. 2a). Gene expression analysis of a panel of 11 well characterised EMT-associated genes was performed by RT-PCR, significant differences were defined as fold change ≥ ±2 and *p*-value ≤ 0.05. In both cell lines, there was a significant increase in expression of the mesenchymal markers, vimentin (VIM) and plasminogen activator inhibitor-1 (PAI-1; Serpine1), while there was a significant decrease in the expression of the epithelial marker E-cadherin (CDH1) (Fig. 2b). Additionally, there was an increase in expression of the EMT associated transcription factor, snail homolog 1 (SNAI1), and signalling protein, jagged 1 (JAG1) (Fig. 2b) in SK-OV-3 cells. In contrast, there was an increase in the mesenchymal marker fibronectin 1 (FN1) with the 59 M cells (Fig. 2b). In line with the previous adhesion and activation data, the platelet effect on SK-OV-3 cells is more pronounced compared to 59 M cells. SK-OV-3 cells appear more responsive to platelet interactions, both in relation to the level and number of EMT-like gene expression changes occurring (Fig. 2b). Overall, these results demonstrate that *in vitro*, platelets promote a more mesenchymal-like phenotype in ovarian cancer cells.

### Platelets significantly enhance the metastatic activity of SK-OV-3 cells

**Fig. 3:**
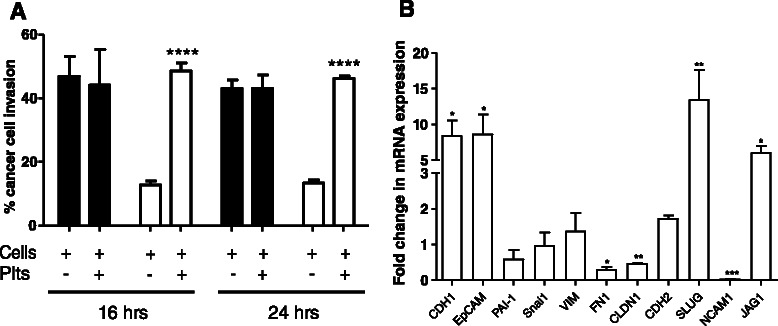
Platelets significantly increase SK-OV-3 cell invasion activity. **a** Results are expressed as the percentage of 59 M (*black bar*) and SK-OV-3 (*white bar*) cell invasion, in the absence and presence of platelets (plts), through Matrigel® invasion chambers relative to control chambers after 16 and 24 h (*n* = 6). **b** SK-OV-3 cells overall have a higher expression level of epithelial markers and lower expression level of mesenchymal markers compared to 59 M. Results are expressed as fold change values that represent the difference in the baseline mRNA expression level of epithelial and mesenchymal markers in SK-OV-3 cells compared to their baseline mRNA expression in 59 M cells (*n* = 3). Values were normalised to beta 2 microglogublin (B2M). Data shown are mean value + SD. ****p ≤ 0.0001, ***p ≤ 0.001, **p ≤ 0.01, *p ≤ 0.05 was determined by Student’s t test

Platelet-induced EMT-like alterations were not only observed at the transcriptome level but also morphologically. To determine whether these changes were of functional importance, we next investigated if platelet cloaked cancer cells had an altered invasive phenotype. Cell lines were incubated alone and with platelets for 24 h, and then allowed to invade in Matrigel® chambers for a further 16 and 24 h. Platelets did not increase the invasive properties of 59 M cells (Fig. 3a). These cells alone had a very high propensity to invade (range: 43–47 % invasion) and therefore it was difficult to assess a direct effect of the addition of platelets on their ability to invade using the Matrigel® invasion assay. In contrast, SK-OV-3 cells had a low invasion activity by themselves (13 % after both 16 and 24 h). Co-culture with platelets resulted in a significant increase in SK-OV-3 cell invasion. Platelets induced a 3.8-fold (*p* = 0.0001) and 3.5-fold (*p* < 0.0001) increase in SK-OV-3 cell invasion after 16 and 24 h, respectively (Fig. 3a). Furthermore, co-culture of SK-OV-3 cells with platelets resulted in a similar high invasion activity to that of 59 M cells alone (49 % versus 47 % after 16 h, and 46 % versus 43 % after 24 h, respectively, Fig. 3a). The dramatically different invasion rates observed between the two cancer cell lines is negated when SK-OV-3 cells are exposed to platelets.

### SK-OV-3 cells have a more epithelial-like phenotype compared to 59 M cells

To identify the molecular mechanism that facilitates high invasion of 59 M cells compared to SK-OV-3 cells in the absence of platelets, we further interrogated the gene expression profile of the 11 selected EMT markers. These results indicate that while both SK-OV-3 and 59 M cells originate from the ascites of patients with metastatic ovarian cancer, their molecular phenotypes are significantly different. SK-OV-3 cells appear to have a more epithelial-like phenotype compared to 59 M cells. This is indicated by the higher expression levels of the epithelial associated markers E-cadherin (CDH1) and epithelial cell adhesion molecule (EpCAM); and lower levels of the mesenchymal markers fibronectin 1 (FN1), claudin (CLDN1) and neural cell adhesion molecule 1 (NCAM1) (Fig. 3b). There are some exceptions to this characterisation with the mesenchymal markers snail homology 2 (SNAI2) and jagged 1 (Jag1) observed to be higher in SK-OV-3 cells. However, the overall pattern of expression, in particular the high expression of epithelial markers, would indicate that SK-OV-3 cells have a more epithelial-like phenotype compared to 59 M cells. This result explains the highly invasive nature of 59 M cells in the absence of platelets.

### Antiplatelet agents significantly reduced the invasion-promoting properties of platelets on SK-OV-3 cells

Having determined that platelets enhance invasion of SK-OV-3 cells, we next investigated if antiplatelet agents effected this interaction. We examined the effect of two widely used antiplatelet therapies: P2Y_12_ inhibition and COX-1 inhibition. Each donor had a RI of ≥70 % in response to each antiplatelet agent, as determined by light transmission aggregometry, and therefore was defined as aspirin- and 2MeSAMP-responders (data not shown).

**Fig. 4:**
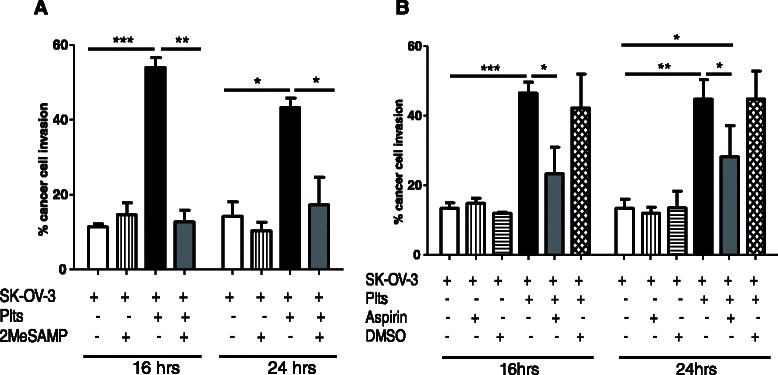
Antiplatelet agents significantly reduce platelet-mediated SK-OV-3 cell invasion. Results are expressed as the percentage of SK-OV-3 cell invasion, in the absence and presence of (**a**) 2MeSAMP-treated platelets (plts) and (**b**) aspirin-treated platelets, through Matrigel® invasion chambers relative to control chambers after 16 and 24 h (*n* = 4). Data shown are mean value + SD. *** *p ≤* 0.001, ** *p ≤* 0.01, * *p ≤* 0.05 was determined by Student’s *t* test

2MeSAMP inhibits platelet function by increasing the levels of cyclic AMP and/or actively competing with ADP for the P2Y_12_ receptor [30]. Upon addition of platelets pre-treated with 50 μM 2MeSAMP, the invasion activity of SK-OV-3 cells significantly decreased 4.3- and 2.5-fold compared to those incubated with untreated platelets after 16 and 24 h respectively (*p* = 0.0065 and *p* = 0.0118, Fig. 4a). Prevention of ADP-induced platelet activation using 2MeSAMP effectively reverted platelet cloaked SK-OV-3 cell invasion to levels comparable to SK-OV-3 cells alone (11 % versus 13 %, and 14 % versus 17 %, after 16 h and 24 h, respectively). No significant changes in SK-OV-3 cell invasion activity were observed when cells were pre-treated with 2MeSAMP alone. Next we studied the effect of aspirin which irreversibly disrupts platelet function by inhibiting the COX-1 enzyme. Exposure of SK-OV-3 cells to platelets pre-treated with 20 μM aspirin resulted in a 2- and 1.6-fold significant reduction in their invasion activity compared to those incubated with untreated platelets after 16 and 24 h respectively (*p* = 0.012 and *p* = 0.0214, respectively, Fig. 4b). In contrast to P2Y_12_ inhibition, although COX-1 inhibition did significantly decrease the platelet-mediated stimulatory effect on the invasion activity of SK-OV-3 cells, it did not completely abolish the platelet effect. When compared with SK-OV-3 cells alone, those exposed to platelets pre-treated with aspirin had a significantly increased invasion activity after both 16 and 24 h (13 % versus 23 %; and 13 % versus 28 %, *p* = 0.028, respectively).

### Platelet adhesion and platelet-induced EMT gene expression alterations are not inhibited by antiplatelet agents

**Fig. 5:**
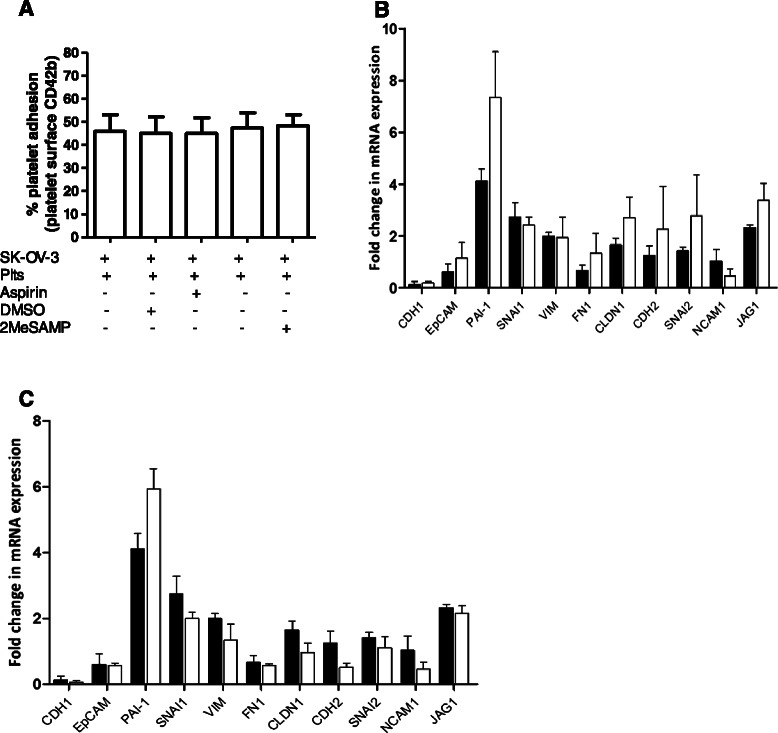
Antiplatelet agents do not affect the ability of platelets to bind to or to induce EMT-like changes in SK-OV-3 cells. **a** Platelet adhesion to SK-OV-3 cells in the presence and absence of aspirin or 2MeSAMP was quantified based on the fluorescence detection of CD42b positive platelets (*n* = 5). **b** Results are expressed as fold change in mRNA expression in SK-OV-3 cells in the presence of platelets (black bar) and platelets pre-treated with 2MeSAMP (*white bar*) relative to cells grown in the absence of platelets for 24 h (*n* = 3). **c** Results are expressed as fold change in mRNA expression in SK-OV-3 cells in the presence of platelets (*black bar*) and platelets pre-treated with aspirin (*white bar*) relative to cells grown in the absence of platelets for 24 h (*n* = 3). Values are normalised to B2M (beta 2 microglobulin). Data shown are mean value + SD. Student’s t-test was performed to determine significant differences between expression levels of the EMT markers in the SK-OV-3 cells cultured with platelets compared with those cultured with platelets pre-treated with antiplatelet agents. No significant differences were identified (significance was defined as *p* ≤ 0.05)

We next sought to determine whether antiplatelet agents could inhibit platelets from cloaking and inducing EMT-like molecular changes in SK-OV-3 cells. The degree of platelet cloaking was measured based on the fluorescent detection of labelled platelets on the surface of cancer cells post incubation. The ability of platelets to adhere to SK-OV-3 cells was not diminished in the presence of either aspirin or 2MeSAMP (Fig. 5a). xFurthermore, analysis of the gene expression profile of the 11 selected EMT markers in SK-OV-3 cells incubated with drug treated and untreated platelets for 24 h were preformed. While pre-treatment of platelets with antiplatelet agents diminishes the platelet-induced invasion capacity of SK-OV-3 cells, it did not inhibit their ability to induce gene expression alterations in EMT-related genes. There were no significant differences in the expression values between SK-OV-3 cells exposed to platelets and those exposed to platelets pre-treated with either antiplatelet agent (Fig. 5b and c).

## Discussion

At initial diagnosis, patients with ovarian cancer often present with widespread metastasis, and this tumour burden constitutes a major clinical management challenge. The biological mechanism driving this route of metastasis remains poorly understood. Platelet interactions with CTCs play a key role in successful haematogenous metastasis. We investigated the interactions between platelets and two metastatic ovarian cancer cells lines, and found them to be significantly heterogeneous. In comparison to 59 M cells, SK-OV-3 cells induced significantly higher levels of both platelet adhesion and activation, two key events in TCIPA. Previous studies have found that there is a direct correlation between the ability of CTCs to interact with platelets via TCIPA, and their increased survival in circulation and subsequent formation of metastatic foci [31, 32]. The ability of SK-OV-3 cells to induce high levels of TCIPA suggests these cells may have a dependency on platelets for initiation and/or enhancement of metastasis.

Although each cell line induced different levels of TCIPA, we found that the direct interaction between platelets and both cancer cells induced similar EMT-like morphological changes. Interestingly, while both cell lines displayed changes from a characteristic epithelial-like morphology to a more spindle, fibroblast-like shape with migratory protrusions, there were distinct differences in their expression profiles. Platelets significantly increased upregulation of JAG1 and SNAI1 in SK-OV-3 cells. JAG1, which encodes a ligand for the Notch receptor, has been shown to induce expression of SNAI2, which along with SNAI1, form part of a key network of transcription factors identified to regulate epithelial-to-mesenchymal plasticity through repression of E-cadherin [33, 34]. Upregulation of these two key mediators of EMT were not observed in the expression profile of 59 M cells cloaked with platelets. During EMT cancer cells have been reported to go through an intermediate phase, where both epithelial and mesenchymal characteristics are present [35, 36]. More recently, SK-OV-3 cells have been described as having an intermediate mesenchymal state because at the protein level these cells are E-cadherin negative and pan-cytokeratin and vimentin positive [37]. Therefore, having observed increased levels of both TCIPA and EMT-like molecular changes in SK-OV-3 cells, compared to 59 M cells, it is reasonable to conclude that SK-OV-3 cells are reliant on platelets to augment their ability to develop a more favourable mesenchymal-like state to maximise their ability metastatic capacity.

Indeed, further to these findings we demonstrated that platelets had a significantly stimulatory effect on the invasive capacity of SK-OV-3 cells. Co-incubation of SK-OV-3 cells and platelets appeared to induce a more aggressive phenotype in these cells. Platelets induced over a 3-fold increase in SK-OV-3 cell invasion at a ratio of 1 cancer cell to 1000 platelets. This ratio is approximately 10–100 times lower than physiological concentrations, suggesting that levels of platelets that promote invasion are present *in vivo*. In contrast, 59 M cells had an intrinsic ability to invade in the absence of platelets. Their interaction with platelets did not augment their invasive capabilities. In fact, platelets appeared to have little stimulatory effect on 59 M cells despite our previous findings that they induce an EMT-like state in these cells. This suggests that platelet interactions with ovarian cancer cells are heterogeneous, both in terms of adhesion and alterations in phenotypic behaviour. The observed difference is most likely due to the finding that SK-OV-3 cells are more epithelial-like at the outset than 59 M cells. Thus, when SK-OV-3 cells come in contact with platelets the effect of transitioning to a more mesenchymal-like state has a more dramatic effect on their overall invasive phenotype. These results support previous findings that cancer cells that have intravasated into the bloodstream without losing their epithelial-like characteristics can acquired a mesenchymal-like phenotype in transit via interaction with platelets [15].

Recently published secondary analyses of cardiovascular trials indicate that daily aspirin reduces not only the short- and long-term risk of death due to cancer, but also distant metastasis by up to 50 % [25–27]. Although multiple mechanisms affecting enzyme activity, transcription factors, cellular signalling and mitochondrial functions have been proposed [38], the chemopreventative effects of aspirin have not been consistently observed in all studies, and thus it is likely that additional mechanisms are involved. More recently, reports have emerged examining the chemopreventative properties of other antiplatelet agent classes, including those targeting ADP receptors P2Y_2_ and P2Y_12_. Studies have shown that P2Y_2_ and P2Y_12_-deficient mice exhibited decreased tumour metastasis [39, 40]. Furthermore, the P2Y_12_ inhibitor ticagrelor was shown to significantly decrease metastasis and improve overall survival in a melanoma mouse model [41]. The finding that platelets increased the ability of SK-OV-3 cells to invade led us to investigate whether this effect could be reversed by pre-treating platelets with antiplatelet agents. During TCIPA, activated platelets secrete several potent factors, including thromboxane A2 and ADP, activated via the COX-1 and P2Y_12_ platelet activation pathways respectively, which in turn heightens the process of activation and aggregation. Our results show that inhibiting these pathways leads to a significant abrogation of invasiveness of SK-OV-3 cells. This effect was particularly evident after exposure of SK-OV-3 cells to platelets that were pre-treated with 2MeSAMP. Prevention of ADP-induced platelet activation using 2MeSAMP effectively reversed invasion to levels comparable to SK-OV-3 cells alone. While a significant decrease in invasion was observed upon exposure to aspirin, levels were still significantly higher compared to SK-OV-3 cells alone. These results suggest that the P2Y_12_ inhibitor 2MeSAMP has a greater inhibitory effect on the ability of SK-OV-3 cells to invade in the bloodstream compared to aspirin.

Upon further molecular interrogation of SK-OV-3 cells exposed to platelets pre-treated with either aspirin or 2MeSAMP, we found that their EMT profile was not altered. These results suggest that neither drug plays a direct role in the regulation of epithelial-to-mesenchymal plasticity, and therefore another mechanism by which they exert their cancer-protective effect is likely. During cancer progression, TCIPA leads to the release of numerous soluble growth factors from alpha granules of activated platelets, which may also prime cancer cells for invasion. For example, the release of PDGF (platelet-derived growth factor), VEGF (vascular endothelial growth factor), uPA (urokinase plasminogen activator) and TGF-β have all been shown to increase the invasive capabilities of cancer cells *in vitro* [15, 42, 43]. Additionally, matrix metalloproteinase (MMP) expression is reportedly increased in the process of TCIPA to facilitate tumour cell migration and invasion [44–46]. MMPs are zinc-dependent endopeptidases that are involved in the degradation of extracellular matrix and are involved in the structural remodelling process. Numerous studies demonstrate that aspirin can inhibit the expression and release of MMP-2 and MMP-9 [47, 48]. Jiang et al. reported that aspirin inhibits MMP-2 activity and *in vitro* invasion of liver cancer cells [49]. Similarly, Tasi et al. demonstrated that aspirin inhibits MMP-2 production and reduces colorectal metastasis in a mouse model [50]. SK-OV-3 cells have been previously shown to secrete both MMP-2 and MMP-9 [51]. Therefore, it is possible that the decreased invasion we observed in our system may be as a direct result of aspirin inhibiting the release of essential MMPs from platelets which aid in degrading the extracellular matrix and subsequent extravasation.

## Conclusions

In conclusion, we demonstrate a new mechanism of platelet-cancer cell interactions. Our results demonstrate that platelet adhesion to ovarian cancer cells transforms them into an EMT phenotype, both functionally and at a molecular level, which is essential for migration, extravasation and formation of distant metastasis. Moreover we demonstrate for the first time that inhibiting platelet function using aspirin and 2MeSAMP disrupts the critical extravasation step in the cascade of metastatic ovarian cancer. As P2Y_12_ inhibitors are now used routinely in the clinic, our data suggests that this class of antiplatelet agent may be a therapeutic option to prevent tumour metastasis in specific cancers.

## Abbreviations

CTCs: Circulating tumour cells
EMT: Epithelial-mesenchymal transition
TGF-β: Transforming growth factor β
2MeSAMP: 2-methylthioadenosine 5′-monophosphate triethylammonium salt hydrate
PRP: Platelet-rich plasma
APC: Allophycocyanin
COX-1: Cyclooxygenase-1
ADP: Adenosine diphosphate
